# Association between Water Fluoride Levels and Low Birth Weight: National Health and Nutrition Examination Survey (NHANES) 2013–2016

**DOI:** 10.3390/ijerph19158956

**Published:** 2022-07-23

**Authors:** Aaditya Krishna Arun, Luis Rustveld, Ajeesh Sunny

**Affiliations:** 1 Harmony School of Innovation, Sugarland, TX 77498, USA; aaditya.arun@bcm.edu; 2 Department of Molecular Cell Biology, Baylor College of Medicine, Houston, TX 77030, USA; 3 Department of Family and Community Medicine, Baylor College of Medicine, Houston, TX 77098, USA; ajeesh.sunny@bcm.edu

**Keywords:** fluoride, NHANES, low birth weight, very low birth weight, Hispanic/Latina paradox

## Abstract

**Background**: Excessive fluoride consumption affects reproductive and child health. We examined the association between levels of fluoride in drinking water and birth weight, in the National Health and Nutrition Examination Survey 2013–2016, after adjusting for known risk factors Low Birth Weight (LBW) including age, smoking, and socio-demographic variables including education, food security, health care access, and health status. **Methods:** The study included 7147 and 6858 women with complete birth weight and water fluoride data, respectively. Linear regression models evaluated the association between water fluoride and birth weight across racial/ethnic groups. The odds of delivering an LBW infant (<2500 g) compared to an infant weighing ≥ 2500 g, as well as the odds of delivering a Very Low Birth Weight (VLBW, <1500 g) infant compared to an LBW infant were explored in separate logistic regression models. **Results:** Women with LBW infants were exposed to significantly higher levels of water fluoride compared to those with normal birth weight infants. Our findings suggest a significant association between excess water fluoride exposure (>0.7 ppm) and LBW weight in Hispanic women, independent of established LBW risk factors. In logistic regression models, Hispanic women exposed to increased levels of water fluoride were 1.5 times more likely to give birth to an LBW infant and 3.5 more likely to give birth to a VLBW infant. **Conclusion:** Taken together, these findings can inform public health education strategies that highlight water fluoride as a potential risk factor during pregnancy in Hispanic women. More research is needed to confirm these findings.

## 1. Introduction

Low Birth Weight (LBW) and Very Low Birth Weight (VLBW) describe infants weighing less than 2500 g and less than 1500 g, at birth, respectively. Both LBW and VLBW [1] children are a heterogeneous group with a wide spectrum of growth, health, and developmental outcomes [2]. Some of these include a higher risk for chronic conditions such as cardiac problems, respiratory problems, neurological issues, and growth deficiencies that last throughout the lifespan of a child, all of which are thought to be exacerbated in VLBW babies [1]. Studies show that LBW is associated with socioeconomic factors such as a mother’s age, familial education, income, and medical history. These socioeconomic factors can aggravate health conditions, specifically neurological and cognitive defects. In addition, factors that contribute to LBW include pregnancy-associated comorbidity, low maternal iron intake, low maternal weight gain during second and third trimesters, and premature birth [3]. Furthermore, environmental factors such as airborne particulate matter, carbon monoxide, and sulfur dioxide content [4], tobacco smoke, heavy metals, pesticides, chlorination byproducts, etc., have been described to be associated with LBW. Similarly, contamination in drinking water has also been described to influence birth weights [5], most of which is organic compounds released from industrial waste [6,7].

Adequate amounts of fluoride are required to mineralize bones and teeth, which is needed to make them strong [8]. Over the past years, fluoride has been added to the community water in the United States and many other countries. Community water fluoridation is supported by multiple organizations, including World Health Organization, Dental Organizations, and US Public Health Services [9]. Fluoridation of tap water started in the 1940s after research in Michigan, New York state, and Ontario province in Canada showed significantly less tooth decay in communities that had access to fluoride in their water. This has since resulted in about 63% of the US having fluoridated water. This was supported by the US Center for Disease Control and Prevention (CDC), highlighting water fluoridation as one of the 10 most significant public health achievements in the 20th century.

Studies have demonstrated that fluoride accumulates primarily in the bone and teeth [10]. High levels of fluoride are also known to accumulate in the calcified regions of the pineal gland [11]. High levels of fluoride in pineal glands have been reported to interfere with melatonin production [12] and hence affect the sleep–wake cycle [13]. In addition, excess fluoride exposure has been shown to cause early onset of puberty in women [14]. Additionally, excess fluoride has been shown to damage various organs and body systems including the nervous system and the skeletal system. Furthermore, excess fluoride exposure has been shown to cause a number of pregnancy-related complications including abortions, congenital abnormalities, anemia, and intra uterine death [15].

A recent commentary in the journal *Nature* discusses the benefits and possible harm caused by fluoride exposure, especially in newborns [16]. Studies in female rats demonstrated a reduction in reproductive hormones, damaged endometrium, reduced ovarian follicles, and a significant reduction in a successful pregnancy, among animals exposed to high concentrations of drinking water sodium fluoride over a 6-month period [17].

The relationship between maternal fluoride exposure and adverse pregnancy outcomes, including the risk of miscarriage, stillbirth, and preterm and LBW infants, has been explored before in areas with excessive water fluoride levels (>1.5 mg/L) [15,18,19]. Alarmingly, a recent study has, for the first time, demonstrated that excess fluoride exposure in utero can lead to babies with low IQ and altered neuronal development [20]. This data came from 512 mother–child pairs in six cities of Canada, suggesting potentially harmful effects of fluoride exposure in babies exposed in utero to this compound. In contrast, other studies showed a protective effect of fluoride exposure against adverse pregnancy outcomes [21,22,23].

A recent study suggested that the benchmark threshold levels for fluoride in the urine of pregnant women should be around 0.2 mg/L [24]. This highlights the potential deleterious role of excess fluoride on fertility and offspring. Although, the exact mechanism involved in fluoride exposure and LBW has not been fully elucidated yet, mounting research has shown that when fluoride is ingested by pregnant women, it can reach the fetus through the umbilical cord and placenta [25,26]. We examined data from the National Health and Nutrition Examination Survey (NHANES) to look for possible associations between excess exposure to fluoride in drinking water and birth weight in a representative sample of the US population. In addition, we also tested the association between excess fluoride (>0.7 ppm, threshold set by the Center for Disease Control and Prevention) [27] and LBW after adjusting for known risk factors associated with LBW, stratified by racial/ethnic group.

Additionally, the motivation to explore the association between water fluoride exposure across racial/ethnic groups stemmed from findings we obtained from a Geographic Information System (GIS) analysis conducted to examine the relationship between low birth weight and fluoride level across areas with the heaviest Hispanic population density in Texas. The GIS maps showed that most Hispanics (>50%) reside in areas with the highest concentration of fluoride in drinking water (1.21–5.10 ppm), in particular, the Northwest regions of Texas. Similarly, the greatest prevalence of LBW (8.7–21.7%) in Texas is observed in areas with the greatest concentration of fluoride in water (unpublished).

## 2. Methods

**Study design and research population:** The National Health and Nutrition Examination Survey (NHANES) is a cross-sectional survey designed and administered by the National Center for Health Statistics (NCHS). Starting in 1999, NHANES is conducted annually and employs a stratified multistage probability design to obtain a nationally representative sample of the civilian, non-institutionalized US population [28]. The survey oversamples Mexican Americans, Non-Hispanic blacks, 12–19-year-old individuals, those older than 60, and low-income individuals. NHANES data are collected by asking participants health-related questions, history of chronic conditions, health-seeking behaviors, substance use, and multiple other risk factors. Participants are invited to come to the Mobile Examination Center (MEC) to obtain several comprehensive physical examinations, including body measurements, blood pressure readings, ultrasound, laboratory tests, spirometry, and dietary interviews. The survey received human subject approval from the National Center for Health Statistics (NCHS) and written informed consent was obtained from each participant before interviews and MEC health exams. A detailed description of the NHANES survey methodology has been published elsewhere [29]. This study did not require ethical approval because NHANES data is open to the public, and the data used in this study had no identifying information. Notably, for our analysis, we did not request access to the NHANES geo-coded data from the NCHS.

Out of a combined 20,146 participants who participated in NHANES between 2013 and 2016, a total of 7147 women had complete infant birth weight data, data on age, education, access to health care, food security, and smoking. From this subgroup, 6858 participants had complete data on water fluoride. Thus, 12,999 subjects were excluded out of a total of 20,146 subjects in the NHANES study (2013–2016) for lack of birth weight data. Furthermore, among, 7147 subjects who had birth weight data, 289 subjects did not have data on fluoride and were hence excluded. Thus, the analytic sample included 6858 subjects.

In the NHANES dataset, all measures included in analysis were obtained using interviewer-administered questionnaires. To conduct subgroup analyses, we combined Mexican American and other Hispanic groups in NHANES into an overall Hispanic category. The age at delivery was used in all the analyses. Low Birth Weight (LBW) and Very Low Birth Weight (VLBW) describe infants weighing less than 2500 g and less than 1500 g, at birth, respectively.

Water consumption and source of water supply in NHANES were collected using 24 h recalls with participants. The source of tap water was ascertained with the question, *“When you drink tap water, what is the main source of the tap water? Is it the city water supply; a well or rain cistern; spring water; or something else?”* The tap water source variable was then coded as “community water supply,” “well or rain cistern,” “spring,” and “don’t drink tap water.” The 24 h recall also included a question on participants’ total plain water consumption the day before the MEC interview. The total plain water variable included consumption of tap water and bottled water.

Water fluoride levels in NHANES were measured in drinking water (in mg/L, also termed parts per million or ppm). Water fluoride samples were then shipped overnight on ice to Georgia Regents University (Augusta, GA, USA) so that fluoride concentration could be measured electrometrically using an ion-specific electrode. Two samples from tap water were obtained from each participant’s residence and were measured for their fluoride levels. The average of these measures was used for the analysis. More details on water fluoride collection methodology can be found in the NHANES laboratory procedure manual [30].

Independent variables were chosen based on established risk factors for LBW documented in the literature and socio-demographic factors [31]. These factors included age, education, overall health status, a routine place for seeking health care, food security, and smoking status.

Education level was obtained with the following question: *what is the highest grade or level of school you completed or the highest degree you received?* Responses included, “less than 9th grade,” 9–11th grade,” “high school grad/GED or equivalent,” “some college or AA degree,” and “college graduate or above.” For the analysis, the Education level “less than 9th grade,” was grouped, and the rest were combined into a common group.

Health status was derived by asking participants whether they could classify their general health condition as: “excellent,” “very good,” “good,” “fair,” or “poor.” For the analyses, we coded the “excellent” and “very good” responses into a “good/excellent” category, and the “fair” and “poor” responses into the “fair/good” category. The “poor” category was used as such.

Having a routine place for seeking health care was asked with the following question: *Is there a place that you usually go to when you are sick or when you need health advice?* Responses were coded as “yes,” “there is no place,” and “there is more than one place.” For the analyses, we combined the responses “yes” and “there is more than one place” into an overall “yes” response, and coded the response ‘there is no place” as “no.”

Food security was ascertained by asking respondents to characterize their overall food security status. Responses were coded as a four-level categorical variable: 1 = full food security; 2 = marginal food security; 3 = low food security; and 4 = very low food security. For the analyses, we combined levels 1 and 2 into a “secure” category, and levels 3 and 4 into a “low food security” category.

Maternal tobacco use was assessed with the question: *did you smoke at any time while pregnant?* Responses were coded as “yes” or “no” and used as such.

Statistical Analysis: SPSS software was used to merge, clean, and recode NHANES variables in preparation for statistical analysis. To achieve adequate sample sizes for subgroup analyses, data from four years of NHANES was combined (2013–2016). NHANES data files are made available to the public in cycles of two years. Two 2-year sample weights were divided by 2 to get the total 4-year sample weight used in analyses (ref). The weights were adjusted for: (1) the different sampling rates for Mexican Americans, African Americans; (2) non-coverage; and (3) non-response bias.

The main objective of the analysis was to explore the association between fluoride levels in drinking water (measured in mg/L) and birth weight (measured in grams) across racial/ethnic groups. Categorical variables were summarized as frequencies and percentages by racial/ethnic groups, and statistically significant differences in proportions were determined using Chi-square tests. Dummy variables were created for all categorical variables (coded as 0, 1) and entered as such as independent variables in linear and logistic regression analyses. Continuous variables were summarized by means and standard errors, and statistically significant differences were determined using analysis of variance (ANOVA). Statistical significance was set at a two-sided significance level of *p* ≤ 0.05.

Due to the skewed distribution of birth weight and water fluoride, we log-transformed these values before inclusion in the linear regression models. Birth weight was considered as an outcome variable in univariate and multivariate regression analyses, and water fluoride was used as the main independent variable. Water fluoride and maternal age at delivery were also entered in all models as continuous variables. Potential confounders were selected and included in regression models based on previous literature, including age, education, overall health status, a routine place for receiving health care, food security, and smoking status. In addition, we explored the association between an interaction term between water fluoride and food security and birth weight in regression models. A categorical birth weight variable was created to include normal birthweight (>2500 g), moderate birth weight (1500–2499 g), and VLBW (<1500 g).

We fit five models to the data: Model 1 presented a univariate linear regression analysis in which water fluoride was entered as the sole independent variable, Model 2 adjusted for maternal age at delivery, Model 3 adjusted Model 2 for education, health status, and routine place for receiving health care, Model 4 adjusted Model 3 for food security, and Model 5 adjusted Model 4 for maternal smoking during pregnancy. Results were expressed as standardized beta coefficients, standard errors, and 95% confidence intervals.

In addition, we also conducted two separate logistic regression analyses to explore the association between water fluoride exposure at >0.7 ppm and birth weight, controlling for age, education, and maternal smoking. In one multivariable logistic regression model, LBW was included as the dependent variable and in the second model, VLBW was the dependent variable (Supplementary Table S1).

## 3. Results

Descriptive Statistics (Table 1): Overall, the sample population included 7147 women who participated in the continuous NHANES study between 2013 and 2016. Of these individuals, 6858 individuals had complete water fluoride data, 27.1% self-classified as Hispanic, 57.5% Non-Hispanic White (NHW), 15.3% Non-Hispanic Black (NHB), and 10.7% other racial/ethnic group. Racial/ethnic characteristics and all the descriptive statistics are presented in Table 1.

NHW and women belonging to other racial/ethnic groups were significantly older than NHB, and Hispanic women (28.5 and 28.9 vs. 25.7 vs. 26.5 years, respectively, *p* <0.001). Significant racial/ethnic disparities were observed in education, access to health care, and food security. Almost 21.9% of Hispanic women did not complete high school education, compared to NHWs, NHBs, and women belonging to other racial/ethnic groups (3.1%, 2.0%, and 6.2%, respectively, *p* < 0.001). Similarly, Hispanic women were more likely to report not having a regular place to obtain health care than NHWs, NHBs, and women belonging to other racial/ethnic groups (5.5%, 3.9%, 2.3%, and 4.3%, respectively, *p* < 0.001). Nevertheless, there was no significant difference in self-reported health status (fair to excellent) across racial/ethnic groups. Hispanics and NHBs were more likely to report food insecurity (33.2% and 26.5%, respectively) compared to 17.4% for NHWs and 16.1% for women belonging to other racial/ethnic groups. Smoking prevalence during pregnancy was also significantly lower among Hispanics, compared to NHW, NHB, and women belonging to other racial/ethnic groups (4.7%, 15.1%, 10.0%, and 9.2%, respectively, *p* < 0.001). As expected, the overall prevalence of LBW (very LBW to moderate LBW combined), was significantly greater for NHB (14.9%) and women belonging to other racial/ethnic groups (11.6%), compared to Hispanic women (8.6%) and NHW (6.8%, *p* < 0.001).

As can be seen in Table 1, community water was the main source of tap water for all racial/ethnic groups (NHW: 77.1%; NHB: 68.1%; Hispanic: 61.2%; and other racial/ethnic groups: 75.3%). The total plain water variable was added as a continuous variable in the univariate model, but it was not significant. We, therefore, did not include it in multivariable models.

Overall, across the entire study group, water fluoride levels were significantly higher in women who gave birth to LBW babies vs. normal birth weight babies (0.53 ± 0.04 vs. 0.49 ± 0.04, *p* = 0.05). Women who gave birth to LBW babies were exposed to comparable mean levels of water fluoride across racial/ethnic groups. However, a significantly higher mean level of water fluoride exposure was observed among Hispanics and other races/ethnic group who gave birth to VLBW babies (0.79 ± 0.62 for Hispanics and for other racial/ethnic groups 0.71 ± 0.37 vs. 0.45 ± 0.35 for NHW vs. 0.57 ± 0.30 for NHBs, *p* = 0.01) (Table 1).

Further analysis using linear regression highlighted a significant association between water fluoride levels and lower birth weight in Hispanic women, but not in NHW and NHB. As shown in Table 2, a univariate linear regression model containing only fluoride as a co-variate or Models 2–5 that included additional risk factors including smoking [32] and socio-demographic variables with fluoride, clearly demonstrated a significant association between water fluoride levels and lower birth weight only in Hispanic women (standardized beta coefficient −0.05 (SE, 0.47) *p* = 0.03, Table 2). In contrast, in both NHW, NHB, and women from other racial/ethnic groups, water fluoride was not associated with LBW after adjusting for covariates in the model. Age was significantly associated with LBW in both NHB and Hispanic women (NHB, standardized beta coefficient 0.07, SE 0.03; Hispanic, standardized beta coefficient 0.06, SE 0.02) in Model 5. Along similar lines, maternal smoking at pregnancy was also associated with LBW in both NHW, Hispanic women, and women belonging to other racial/ethnic groups (NHW, standardized beta coefficient 0.11, SE 0.36, *p* < 0.001; Hispanic, standardized beta coefficient 0.08, SE 0.62, *p* < 0.001; and women belonging to other racial/ethnic groups, standardized beta coefficient 0.07, SE 0.67, *p* = 0.03). In only NHW but not in the other groups, both education and place to receive health care were significantly associated with LBW (Table 2, Models 3–5). Along similar lines, only in NHB (Models 3–5) and women belonging to other racial/ethnic groups (Model 4), poor health was significantly associated with LBW (Table 2).

The interaction term between water fluoride and food security was not significant in the univariate linear regression model across racial/ethnic groups. We, therefore, used the original food security variable in all analyses. Results from the logistic regression analysis are shown in Supplementary Table S1 as three separate multivariable logistic regression models. In Model 1, water fluoride exposure levels > 0.7 ppm (0.7 ppm is the safe threshold set by CDC) was significantly associated with LBW (Hispanic: OR 1.49, 95% CI 1.13, 1.97, *p* = 0.005; NHW: OR 1.01, 95% CI 0.71, 1.43, *p* = 0.10; NHB: OR 1.12, 95% CI 0.86, 1.45, *p* = 0.41; other racial/ethnic groups: OR 1.43, 95% CI 0.98, 2.08, *p* = 0.06) after adjusting for mother’s age at delivery, education < 9th grade and smoking during pregnancy. Since published data describe the increasing severity of chronic disease in children who have very low birth weight (VLBW, <1500 g), we examined if exposure to fluoride during pregnancy results in VLBW infants compared to both normal birth weight and LBW infants. In these comparisons (Models 2 and 3), Hispanic women exposed to fluoride levels > 0.7 ppm were 3.5 times more likely to deliver VLBW (*p* < 0.001, Supplementary Table S1) babies. It was not possible to examine the association between smoking and VLBW in women of other racial/ethnic groups, due to small sample size.

Taken together, our analysis describes a significant association between excess water fluoride exposure and LBW in Hispanic women. In addition, exposure to excess water fluoride significantly increases the odds of Hispanic women delivering a VLBW infant.

## 4. Discussion

Our findings, using population-based data from NHANES, suggest a significant association between excess water fluoride exposure (>0.7 ppm) and LBW weight, independent of established LBW risk factors. Our results concur with findings from Ortíz-García and colleagues, who examined the impact of fluoride exposure in pregnancy and birth weight, in the Early Life Exposures in Mexico to Environmental Toxicants (ELEMENT) cohort study. The ELEMENT study is a prospective cohort study designed to examine the impact of environmental toxicants on maternal and child health in Mexico. The authors concluded that prenatal fluoride exposure was significantly associated with a decrease in birth weight starting with incremental levels of fluoride exposure in the second and third trimesters (β = −0.25; 95% CI: −0.55; 0.04, *p* = 0.09 and β = −0.33; 95% CI: −0.63, −0.03; *p* = 0.03) [33]. An important consideration in comparing findings between the ELEMENT study and ours is that it included a more homogeneous population of women of Mexican descent, as data is collected during pregnancy or delivery from maternity hospitals in Mexico City, Mexico. In contrast, NHANES collects very detailed information on Hispanic ethnic origins, which are then classified under the Hispanic or Latino category. Hispanic ethnicity groups include Mexican, Mexican American, Chicano, Puerto Rican, Cuban, Cuban American, Dominican, Central or South American. Our results are, therefore, generalizable across the US population. Additionally, per our understanding of the ELEMENT study, the water fluoride levels were not as variable as those found in the NHANES study conducted in the United States. Water fluoride samples taken in several urban and rural areas where the ELEMENT study cohort reside, indicate that water fluoride levels in Mexico City can range from 0.15 to 1.38 mg/L [34]. In the US, it is common practice for fluoride to be added to drinking water at levels of 0.7–1.2 mg/L [35]. The NHANES study contained subjects who were exposed to very high water fluoride levels beyond the acceptable threshold set by Environment Protection Agency. As can be seen in Table 1, community water was the main source of tap water for all racial/ethnic groups.

The observed significant association between excess fluoride and LBW in Hispanic women contrasts with existing epidemiological evidence, suggesting that Hispanic women are more likely to give birth to normal-weight babies, as described in the literature as the Latina epidemiologic paradox [36]. The Latina epidemiologic paradox contends that even when taking into consideration health inequities, Latina women are less likely than non-Latina women to give birth to an LBW baby [37,38,39]. Potential explanations for the observed lower prevalence of LBW babies among Latina women have varied widely and included sociocultural factors such as spirituality [40], social support [41], and discrimination [42,43]. Our findings on NHB women also corroborates previous findings that place African American women at significant risk of delivering an LBW infant [44]. However, excessive fluoride exposure in our study did not affect the LBW prevalence in NHB women.

Children who weigh < 2500 g at birth are known to be predisposed to several chronic diseases, including diabetes, cognitive impairments, cardiovascular, metabolic disorders, and even mortality, either during childhood or early in their adult life, with the severity of the condition predicted to be more pronounced for VLBW babies [1]. In the United States, VLBW accounts for approximately 1.0% of live births. Necrotizing enterocolitis, intraventricular hemorrhage, bronchopulmonary diseases, and retinopathy are linked to VLBW [1]. Our findings indicate that NHBs are almost 2.4 times more likely to give birth to a VLBW baby compared to NHW (2.4% vs. 0.7%, respectively), followed by Hispanics (1.5%). Our findings are also consistent with a report published by Martin JA and colleagues [45], where the authors examined fertility patterns and maternal characteristics in birth certificates of 3.95 million births that occurred in 2016. They reported that VLBW was more prevalent in NHB compared to NHW and Hispanic women (2.95%, 1.07%, and 1.24%, respectively) [45].

Evidence suggests that inadequate maternal nutrition during pregnancy contributes to LBW and intrauterine growth restriction [46,47]. We examined food security as a proxy for maternal nutrition status, to determine its impact on birth weight. A study undertaken on 881 pregnant adolescents in New York reported that over half the subjects reported food insecurity and this was further associated with LBW and early gestational age [48]. In an independent study conducted in Bangladesh, mothers who had food insecurity had about 38% higher likelihood of having smaller sized babies compared to mothers who had food security [49]. In yet another facility based unmatched case–control study, food insecurity was one among many factors associated with LBW babies [50]. A similar study in Malawian women further demonstrated a strong association between food insecurity for mothers and LBW babies [51].

Interestingly, although the mean fluoride levels were comparable across racial/ethnic groups, we observed a significantly higher mean for water fluoride in Hispanic women who gave birth to VLBW babies. Furthermore, water fluoride exposure at 0.7 ppm in a multivariable logistic regression analysis showed a 3.5 times higher likelihood of Hispanic women giving birth to VLBW babies (Supplementary Table S1). The odds of VLBW babies in NHW and NHB in this analysis was not significant. These results have significant public health implications, as fluoride in water can be viewed as a modifiable risk factor. Notably, reduction in excess fluoride can be achieved using simple water filtration devices [52].

Unlike many publicly available datasets, the demographic distribution of the NHANES data represents the US population, and therefore findings are more likely to be generalizable. Several limitations for this study are worth mentioning. Due to the study’s cross-sectional nature, the temporal relationship between water fluoride exposure and LBW cannot be conclusively established. It is unclear whether the women included in this study resided in the same areas with the same water fluoride levels during pregnancy and childbirth as when they participated in the NHANES study. Furthermore, as described in our results, community water was the main source of tap water for all racial/ethnic groups. The limitation, however, is that the water variable included both tap water and bottled water. Therefore, it is difficult to tease out participants who drank only bottled water from those who drank only tap water, because those who drank bottled water may also have drunk tap water. Additionally, no fluoride measures were obtained from bottled water. Nevertheless, in our analysis, the total plain water variable was added as a continuous variable in the univariate model and was not significant across the racial/ethnic groups. We, therefore, did not include it in multivariable models.

We also appreciate that other risk factors in addition to fluoride exposed before and during pregnancy could be related to infants’ birth weights. However, to our knowledge, a systematic study measuring these risk factors and environmental exposures such as water fluoride levels before pregnancy and following it up during pregnancy, has not been undertaken. Specifically, we believe that a study measuring fluoride levels before pregnancy and during pregnancy (preferably every trimester) compared to a control group where fluoride levels in water are kept within acceptable limits both before pregnancy and during pregnancy, may provide useful insights into the direct effect of fluoride in pregnancy outcomes.

In our analysis, we adjusted for a limited set of important variables that we understand is not all-encompassing due to lack of available data points in NHANES. Given this, we included variables that have been consistently collected for the time frame for which LBW data was available. Since all the variables included in the analysis were collected uniformly across all years (2013–2016), we do not believe the pooling could have introduced a bias in our analysis. Importantly, our analysis took into consideration all the samples that had complete data on birth weight and water fluoride levels without any preferential bias to any specific racial/ethnic groups. A follow-up to our original observation showing a significant association between water fluoride and LBW across the entire study population led to additional subset analysis examining race/ethnic groups. Such an analysis revealed that the association between water fluoride and LBW was more pronounced in Hispanic populations.

We also must take into consideration that social desirability bias related to smoking and pregnancy may have resulted in under reporting smoking prevalence during pregnancy. While NHANES collects detailed data on dental practices and fluoride exposure, birth weight data is unavailable for the same years. Therefore, the study could not examine the impact of dental practices, including fluoride exposure from dental sealants and fluoride supplementation. Furthermore, the NHANES was not methodologically designed to examine the association between fluoride exposure and birth weight.

Nevertheless, keeping all these limitations in context, our findings form the steppingstone for longitudinal studies in the future to examine the association of fluoride with LBW across racial/ethnic groups in the US. It is also important to note that in no way do the findings in this study minimize the significant burden of LBW among NHBs, which was observed by us in the context of socio-economic variables.

## 5. Conclusions

Exposure to high fluoride concentrations in water changes the previously observed favorable pregnancy outcome in Hispanic women. Results from this study suggest that exposure to the high concentration of fluoride in drinking water puts Hispanic women at increased risk of delivering an LBW infant. These findings highlight the need for increased attention to the potential contribution of fluoride as an environmental toxicant in fetal development. More research is needed to confirm these findings.
**What is already known on this topic** Excess exposure to fluoride in drinking water significantly affects reproductive outcomes in animal models and human subjects. **What this study adds** This study shows an association between fluoride exposure in drinking water and the likelihood of having a low birth weight baby among Hispanic women. **How this study might affect research, practice, or policy** Findings from this research highlight the need for increased attention to the contribution of fluoride as a potential environmental toxicant in fetal development.


## Figures and Tables

**Table 1:** Characteristics of study participants by racial/ethnic group: NHANES 2013–2016.

Variable	Overall Sample	NHW	NHB	Hispanic	Other	*p*
N (%)	7147	2016 (51.4)	1676 (13.7)	2363 (24.3)	1092 (10.7)	
Maternal Age at Delivery, Mean (SE)	27.1 ± 0.23	28.5 ± 0.30	25.7 ± 0.32	26.5 ± 0.20	28.9 ± 0.39	<0.001
Water Fluoride, Mean (SE)	0.50 ± 0.04	0.46 ± 0.04	0.56 ± 0.03	0.55 ± 0.05	0.48 ± 0.04	<0.001
**Water Supply Source**, Total (%)						<0.001
Community water	3552 (72.0)	1184 (77.1)	810 (68.1)	1009 (61.2)	549 (75.3)	
Well/Rain cistern	313 (8.0)	189 (75.0)	21 (2.5)	78 (5.4)	25 (4.1)	
Spring	65 (1.0)	18 (0.9)	15 (0.7)	21 (1.3)	11 (1.2)	
Do not drink tap water	1412 (19.0)	244 (10.7)	366 (28.7)	650 (32.1)	152 (19.4)	
Birth Weight Group (BWGR), Total (%)						<0.001
>2500 g (Normal BW)	5466 (91.1)	1879 (93.2)	1427 (85.1)	2160 (91.4)	965 (88.4)	
1500–2499 g (Moderate LBW)	503 (7.7)	123 (6.1)	211 (12.6)	169 (7.2)	113 (10.3)	
<1500 g (Very LBW)	87 (1.2)	15 (0.7)	38 (2.3)	34 (1.4)	14 (1.3)	
Water Fluoride (mg/L) by BWGR, Mean (SD)						
>2500 g (Normal BW),	0.52 ± 0.38	0.45 ± 0.35	0.56 ± 0.33	0.56 ± 0.42	0.48 ± 0.33	ns
≤2500 g (LBW)	0.54 ± 0.36	0.44 ± 0.31	0.55 ± 0.31	0.56 ± 0.44	0.54 ± 0.35	ns
<1500 g (Very LBW)	0.65 ± 0.46	0.45 ± 0.35	0.57 ± 0.30	0.79 ± 0.62	0.71 ± 0.37	0.01
Education						<0.001
9–11th grade	1005 (14.1)	186 (9.3)	273 (16.3)	482 (20.7)	64 (5.9)	
High School/GED	1550 (21.8)	382 (19.0)	437 (26.0)	525 (22.5)	206 (18.9)	
Some College/Associate Degree	2253 (31.7)	727 (36.2)	672 (40.0)	565 (24.2)	289 (26.5)	
College Graduate or above	1625 (22.9)	651 (32.4)	263 (15.7)	248 (10.6)	463 (42.5)	
Health Status, Total (%)						<0.001
Good/Excellent	5475 (74.4)	1772 (85.7)	1297 (75.5)	1520 (61.7)	886 (77.9)	
Fair/Good	1853 (25.2)	292 (14.1)	410 (23.9)	907 (37.2)	244 (21.5)	
Poor	35 (0.5)	3 (0.1)	11 (0.6)	14 (0.6)	7 (0.6)	
Health Care Place, Total (%)						<0.001
Yes	7059 (95.9)	1986 (96.1)	1678 (97.7)	2307 (94.2)	1088 (95.7)	
No	304 (4.1)	81 (3.9)	40 (2.3)	134 (5.5)	49 (4.3)	
Food Security, Total (%)						<0.001
Secure	5450 (75.5)	1689 (82.6)	1250 (73.5)	1581 (66.8)	930 (83.9)	
Low	1772 (24.5)	357 (17.4)	451 (26.5)	787 (33.2)	179 (16.1)	
Smoking when Pregnant, Total (%)						<0.001
Yes	695 (9.5)	310 (15.1)	170 (10.0)	113 (4.7)	102 (9.2)	
No	6589 (90.5)	1745 (84.9)	1528 (90.0)	2309 (95.3)	1007 (90.8)	

All percentages are weighted according to the National Health and Nutrition Examination Survey (NHANES) statistical weighting scheme. SD = Standard Deviation; BW = Birth Weight; LBW = Low Birth Weight; Very LBW = Very Low Birth Weight; NHW = Non-Hispanic White; NHB = Non-Hispanic Black; GED = General Education Development.

**Table 2:** Associations between socio-demographic, smoking, and water fluoridation levels and birth weight: data from the National Health and Nutrition Examination Survey (NHANES) 2013–2016.

	Non-Hispanic White				Non-Hispanic Black				Hispanic				Other Races			
	Beta	SE	95% CI	*p*	Beta	SE	95% CI	*p*	Beta	SE	95% CI	*p*	Beta	SE	95% CI	*p*
**Model 1**																
Water Fluoride	−0.02	0.44	−1.14, 0.58	0.52	0.03	0.64	−0.46, 2.08	0.21	−0.05	0.45	−1.96, −0.16	0.02	−0.06	0.67	−2.49, 0.14	0.08
**Model 2**																
Age	0.01	0.02	−0.03, 0.05	0.61	**0.07**	0.02	0.02, 0.12	**0.01**	**0.06**	0.02	0.01, 0.08	**0.01**	0.002	0.03	−0.06, 0.06	0.96
Water Fluoride	−0.02	0.44	−1.17, 0.56	0.49	0.03	0.67	−0.62, 2.10	0.30	**−0.05**	0.45	−1.85, −0.06	**0.03**	−0.05	0.67	−2.43, 0.23	0.10
**Model 3**																
Age	0.02	0.02	−0.03, 0.05	0.52	**0.06**	0.03	0.01, 0.11	**0.01**	**0.06**	0.02	0.01, −0.09	**0.01**	0.01	0.03	−0.06, 0.06	0.99
Water Fluoride	−0.004	0.45	−0.96, 0.81	0.87	0.03	0.68	−0.50, 2.17	0.22	**−0.04**	0.47	−1.86, −0.03	**0.04**	−0.06	0.70	−2.60, 0.13	0.08
<9th-Grade Education	**0.05**	0.78	0.09, 3.14	**0.04**	0.02	1.12	−1.25, 3.07	0.45	−0.01	0.31	−0.70, 0.50	0.74	0.02	0.75	−0.99, 1.93	0.53
Poor Health	−0.04	0.34	−1.29, 0.05	0.07	**−0.07**	0.36	−1.68, −0.26	**0.01**	−0.02	0.26	−0.77, 0.25	0.32	−0.06	0.44	−1.69, 0.03	0.06
Health Care Place	**0.07**	0.63	0.57, 3.04	**0.04**	0.004	1.15	−2.05, 2.45	0.87	−0.003	0.53	−1.10, 0.96	0.89	0.01	0.92	−1.58, 2.03	0.81
**Model 4**																
Age	0.01	0.02	−0.03, 0.05	0.56	**0.06**	0.03	0.01, 0.11	**0.01**	**0.06**	0.02	0.01, 0.09	**0.01**	0.01	0.03	−0.06, 0.07	0.87
Water Fluoride	−0.01	0.45	−0.98, 0.79	0.83	0.03	0.68	−0.50, 2.17	0.22	**−0.05**	0.47	−1.95, −0.09	**0.02**	−0.05	0.70	−2.532, 0.23	0.10
<9th-Grade Education	**0.05**	0.78	0.08, 3.13	**0.04**	0.02	1.13	−1.39, 3.04	0.46	−0.01	0.31	−0.73, 0.50	0.73	0.02	0.78	−1.16, 1.89	0.64
Poor Health	−0.04	0.35	−1.32, −0.03	0.06	**−0.07**	0.37	−1.71, −0.28	**0.01**	−0.02	0.27	−0.76, 0.28	0.36	**−0.06**	0.44	−1.73, 0.001	**0.05**
Health Care Place	**0.07**	0.63	0.51, 2.98	**0.01**	0.001	1.17	−2.25, 2.32	0.98	−0.001	0.55	−1.10, 1.04	0.95	0.01	0.92	−1.58, 2.03	0.81
Food Security	−0.03	0.35	−1.11, 0.26	0.23	0.01	0.35	−0.51, 0.88	0.60	−0.01	0.27	−0.63, 0.43	0.70	0.04	0.50	−0.42, 1.55	0.26
**Model 5**																
Age	−0.004	0.02	−0.04, 0.04	0.88	**0.07**	0.03	0.02, 0.11	**0.01**	**0.06**	0.02	0.01, 0.09	**0.01**	−0.01	0.03	−0.07, 0.06	0.88
Water Fluoride	−0.04	0.45	−0.95, 0.81	0.88	0.04	0.68	−0.40, 2.26	0.17	**−0.05**	0.47	−1.95, −0.09	**0.03**	−0.06	0.71	−2.66, 0.11	0.07
<9th-Grade Education	**0.05**	0.78	0.06, 3.10	**0.04**	0.02	1.12	−1.49, 2.91	0.53	−0.01	0.31	−0.74, 0.48	0.67	0.01	0.78	−1.22, 1.84	0.69
Poor Health	−0.04	0.35	−1.22, 0.14	0.12	**−0.07**	0.36	−1.73, −0.31	**0.01**	−0.03	0.27	−0.81, 0.23	0.27	−0.06	0.44	−1.70, 0.03	0.06
Health Care Place	**0.07**	0.63	0.52, 2.97	**0.01**	−0.01	1.18	−2.77, 1.86	0.70	0.001	0.54	−1.04, 1.01	0.96	0.01	0.92	−1.44, 2.18	0.69
Food Security	−0.02	0.35	−0.95, 0.42	0.45	0.02	0.35	−0.39, 0.98	0.40	0.00	0.27	−0.53, 0.53	0.98	0.05	0.51	−0.27, 1.72	0.16
Smoking	**0.11**	0.36	0.87, 2.26	**<0.001**	0.04	0.55	−0.23, 1.92	0.12	**0.08**	0.62	0.98, 3.41	**<0.001**	**0.07**	0.67	0.19, 2.84	**0.03**

Abbreviations: CI, confidence intervals, SE, standard error of the coefficient. Beta is standardized. Significant coefficients are bolded. Birth weight was the dependent variable in all models. Water fluoride level was log-transformed in all regression analyses. Age is the mother’s age at delivery. All *p*-values are presented after Bonferroni correction to account for multiple comparisons. Linear regression analyses were conducted using NHANES Mobile Exam Center weights.

## Data Availability

NHANES data is open to the public.

## Supplementary Materials

The following supporting information can be downloaded at: https://www.mdpi.com/article/10.3390/ijerph19158956/s1, Table S1: Logistic Regression showing the association between Water Fluoride and Low Birth Weight and Very Low Birth Weight Across Racial/Ethnic Groups: National Health and Nutrition Examination Survey (NHANES), 2013–2016.
