# Supplementary material for: Association between Water Fluoride Levels and Low Birth Weight: National Health and Nutrition Examination Survey (NHANES) 2013–2016
Source: Int J Environ Res Public Health. 2022 Jul 23;19(15):8956. doi: 10.3390/ijerph19158956 (PMC9332356; doi:10.3390/ijerph19158956)
Supplement: Supplementary file 1 [file ijerph-19-08956-s001.zip › ijerph-1789884-supplementary.pdf]

Supplemental Table S1. Logistic Regression showing the association between Water Fluoride and Low Birth Weight and Very Low Birth Weight Across Racial/Ethnic Groups: National Health and Nutrition Examination Survey (NHANES), 2013-2016.

	**Hispanic**			**Non-Hispanic White**			**Non-Hispanic Black**			**Other Race**		
	**OR**	**95% CI**	**p**	**OR**	**95% CI**	**p**	**OR**	**95% CI**	**p**	**OR**	**95% CI**	**p**
**Model 1: Odds of LBW (<2500 grams), ref = Normal Birth Weight (≥2500 grams)**												
Water Fluoride > 0.7 ppm	1.49	1.13, 1.97	0.005	1.01	0.71, 1.43	0.10	1.12	0.86, 1.45	0.41	1.43	0.98, 2.08	0.06
**Model 2: Odds of VLBW (<1500 grams), ref = Normal Birth Weight (≥2500 grams)**												
Water Fluoride > 0.7 ppm	3.50	1.66, 7.17	<0.001	1.80	0.49, 6.30	0.38	1.42	0.73, 2.79	0.30	2.42	0.81, 7.02	0.10
**Model 3: Odds of VLBW (<1500 grams), ref = LBW (<2500 grams)**												
Water Fluoride > 0.7 ppm	3.60	1.55, 8.33	0.003	1.30	0.31, 5.33	0.10	1.29	0.63, 2.65	0.49	1.81	0.57, 5.74	0.31

Abbreviations: OR, Odds Ratio; CI, Confidence Intervals; LBW, Low Birth Weight; VLBW, Very Low Birth Weight, Water fluoride was a categorical variable and > 0.7 ppm. All models adjusted for mother's age at delivery, education <9th grade, and smoking during pregnancy
